# ApoB48 as an Efficient Regulator of Intestinal Lipid Transport

**DOI:** 10.3389/fphys.2020.00796

**Published:** 2020-07-08

**Authors:** Chunmin C. Lo, Karen T. Coschigano

**Affiliations:** The Diabetes Institute, Interdisciplinary Program in Molecular and Cellular Biology, and Department of Biomedical Sciences, Heritage College of Osteopathic Medicine, Ohio University, Athens, OH, United States

**Keywords:** Apobec-1 enzyme, apolipoprotein B, chylomicron, lymph, synthesis, degradation

## Abstract

Fatty meals induce intestinal secretion of chylomicrons (CMs) containing apolipoprotein (Apo) B48. These CMs travel *via* the lymphatic system before entering the circulation. ApoB48 is produced after post-transcriptional RNA modification by Apobec-1 editing enzyme, exclusively in the small intestine of humans and most other mammals. In contrast, in the liver where Apobec-1 editing enzyme is not expressed (except in rats and mice), the unedited transcript encodes a larger protein, ApoB100, which is used in the formation of very low-density lipoproteins (VLDL) to transport liver-synthesized fat to peripheral tissues. Apobec-1 knockout (KO) mice lack the ability to perform ApoB RNA editing, and thus, express ApoB100 in the intestine. These mice, maintained on either a chow diet or high fat diet, have body weight gain and food intake comparable to their wildtype (WT) counterparts on the respective diet; however, they secrete larger triglyceride (TG)-rich lipoprotein particles and at a slower rate than the WT mice. Using a lymph fistula model, we demonstrated that Apobec-1 KO mice also produced fewer CMs and exhibited reduced lymphatic transport of TG in response to duodenal infusion of TG at a moderate dose; in contrast, the Apobec-1 KO and WT mice had similar lymphatic transport of TG when they received a high dose of TG. Thus, the smaller, energy-saving ApoB48 appears to play a superior role in comparison with ApoB100 in the control of intestinal lipid transport in response to dietary lipid intake, at least at low to moderate lipid levels.

## Introduction

Obesity has become a global epidemic, affecting more than 39.8% of adults in the US and increasing risk of cardiovascular diseases (CVD) and stroke (Carbone et al., 2019). Epidemiological investigation demonstrates that hypertriglyceridemia is a causal risk factor for CVD (Sarwar et al., 2010; Do et al., 2013; Jørgensen et al., 2013). Postprandial hypertriglyceridemia leads to coronary artery disease observed in insulin resistance and Type 2 diabetes (Taskinen, 2003; Taskinen et al., 2011). Patients with coronary artery disease and Type 2 diabetes have impaired clearance of apolipoprotein (Apo) B48-containing chylomicrons (CMs) and higher plasma level of ApoB48 and triglyceride (TG) than people without these diseases (Masuda et al., 2012; Mancera-Romero et al., 2013; Tian et al., 2019). In healthy humans, and also mammals where many studies are performed, ApoB48 serves as the main structural apolipoprotein of TG-rich lipoproteins for transporting dietary lipids from the small intestine to peripheral tissues, particularly adipose tissue and the skeletal muscle (Tso and Balint, 1986; Kohan et al., 2015). These dietary lipids are shunted into peripheral tissues to provide energy and to protect the liver from excessive accumulation of TG and/or precursors of TG (Xiao et al., 2011). After uptake of fatty acid (FA) by adipose and muscle cells, CM remnants are taken up by the liver (Xiao et al., 2011). In obese and insulin-resistant subjects, an increase in intestinal production and a reduction in catabolism of postprandial TG-rich lipoproteins lead to hypertriglyceridemia (Ginsberg and Fisher, 2009). Impaired clearance of TG-rich lipoproteins by the liver is related to the accumulation of their remnants in postprandial serum, and the incorporation of these remnants to the arterial wall leads to atherosclerotic lesions (Masuda et al., 2011; Fogelstrand and Borén, 2012). The mechanism underlying clearance of TG-rich lipoproteins and their remnants, where ApoB48 may play an important role, has been studied by multiple investigators (Masuda et al., 2011; Foley et al., 2013; Damsteegt et al., 2018). However, fewer studies regarding the regulation of intestinal TG-rich lipoprotein production have been reported. This perspective focuses on the importance of ApoB48 in the control of intestinal lipid transport.

## Intestinal Lipid Digestion and Absorption

When dietary TG arrives in the intestinal lumen, pancreatic lipase acts on it to release two FAs and 2-monoglyceride (Mattson and Volpenhein, 1964). Subsequently, the released FAs and monoglyceride are absorbed by intestinal enterocytes by either passive diffusion across a concentration gradient or a carrier-mediated process (Dash et al., 2015; Xiao et al., 2019). Fatty acid translocase/cluster of differentiation 36 (FAT/CD36), fatty acid transport protein 4 (FATP4), and fatty acid binding proteins (FABP) in the plasma membranes are transporters that have been implicated in intestinal uptake, especially of long chain fatty acids (Storch and Thumser, 2000; Nauli et al., 2006; Xiao et al., 2019). Once taken up by the enterocytes, monoglycerides and free FAs are recombined to form TG at the endoplasmic reticulum (ER) membrane leaflet. Phosphatidylcholine, the major form of dietary phospholipids, is digested by pancreatic phospholipase A_2_ to yield lysophosphatidylcholine and FA in the intestinal lumen. After enterocyte uptake, most of the lysophosphatidylcholine is reconstituted to form phosphatidylcholine (Cohn et al., 2010). Dietary cholesteryl ester (CE) is hydrolyzed by cholesterol esterase to form free cholesterol in the intestinal lumen before absorption into the enterocyte and re-esterification back to cholesteryl ester; absorption and delivery to the ER is likely facilitated by the Niemann-Pick C1-like 1 receptor (Altmann et al., 2004; Sané et al., 2006; Cohn et al., 2010).

## Apob Production and Cm Assembly and Secretion

In the intestine of humans and other mammals, the mRNA transcript from the *APOB* gene is post-transcriptionally edited in the nucleus of enterocytes by Apobec-1, an enzyme that changes a specific cytidine to a uridine by hydrolytic deamination and introduces a stop codon (Teng et al., 1993; Lau et al., 1994). Apobec-1 is directed to the proper cytidine in part by an auxiliary factor called Apobec complementation factor, which recognizes and attaches to a specific cis-element downstream of the cytidine, called a mooring sequence, and positions Apobec-1 over the correct cytidine for deamination. Translation of the edited RNA results in a truncated ApoB protein called ApoB48. In the liver of humans and most other mammals (except rats and mice), Apobec-1 activity is absent and ApoB RNA editing does not occur, resulting in translation of the full-length ApoB protein, ApoB100 (Damsteegt et al., 2018).

Within the intestinal enterocytes, each ApoB48 is synthesized on a ribosome attached to the ER and translocated through the ER membrane, where it is co-translationally combined with TG, phospholipid, and CE by microsomal triglyceride transfer protein (MTP) and then combined with a B48-free lipid droplet to form a pre-CM (Xiao et al., 2019). Whereas the majority of TGs will be packaged into pre-CMs, a portion of lipids are stored as cytosolic lipid droplets (CLD) that serve as transient storage of TG (Xiao et al., 2020). After addition of ApoA-IV, exit of the pre-CM from the ER is the rate-limiting step in the transcellular movement of the absorbed FA from apical membrane to basal membrane as the CM (Xiao et al., 2019). Pre-CMs packaged in pre-CM transport vehicles (PCTV) are transported from the ER to the Golgi and become mature CMs through addition of ApoA-I, also transferred from the ER, and glycosylation of ApoB48 (Mansbach and Siddiqi, 2016; Xiao et al., 2019). Mature TG-rich lipoproteins exit the enterocyte through the basolateral membrane, pass through the lamina propria by convective movement of fluids, and are taken up by lacteals based on size and composition as well as molecular signaling pathways (Kohan et al., 2015; Xiao et al., 2019). The lacteals drain into the mesenteric lymphatic duct and then the thoracic duct, and eventually, TG-rich lipoproteins enter the venous circulation. Overall, total lymph flow from the intestine to the circulation is achieved in part through a unidirectional and contractile pumping mechanism controlled by the autonomic nervous system and smooth muscle fibers surrounding the lymphatic system (Xiao et al., 2020).

## Regulation of Tg-Rich Lipoprotein Production

Intestinal TG-rich lipoproteins are essential for transporting dietary fats into the circulation (Kohan et al., 2015; Xiao et al., 2019). The secretion of intestinal lipoproteins depends on the physiologic state of the small intestine, e.g., fasting or active fat absorption. The TG-rich lipoproteins made by the small intestine during fasting are small [exclusively very low-density lipoproteins (VLDL)-sized lipoproteins] and become larger during lipid absorption (CM-sized lipoproteins; Tso and Balint, 1986).

In the liver, when MTP activity is low or lipid availability is reduced, such as in a fasting state, excess ApoB100 molecules are co- or post-translationally tagged with ubiquitin and diverted to the proteasome-mediated ER-associated degradation (ERAD) pathway for destruction (Ginsberg and Fisher, 2009; Olofsson and Borén, 2012). When the availability of TG increases and MTP activity is high, less hepatic ApoB100 is degraded in the ER and instead more is assembled into VLDL-sized particles and secreted, with each particle containing one ApoB100 molecule; thus, the number of secreted particles increases with little change in particle size (Elovson et al., 1988; Ginsberg and Fisher, 2009). Several metabolic circumstances, such as exposure to certain dietary polyunsaturated FAs, can lead to ApoB100 degradation *via* a nonproteasomal, post-ER, presecretory proteolysis (PERPP) pathway, likely involving autophagy, in conditions when TG availability and incorporation into VLDL are normal (Ginsberg and Fisher, 2009; Olofsson and Borén, 2012).

In the intestine, the situation is less clear. Based on studies of proteasomal degradation performed in colon cell lines, including HT29 cells (a human colon adenocarcinoma cell line) and CaCo_2_ cells (a human colon carcinoma cell line), little or no ubiquitination and proteasomal degradation of ApoB48 synthesized in colon cell lines is observed (Liao and Chan, 2000; Dixon et al., 2002). In contrast, another group reported ApoB48 degradation in CaCo_2_ cells and primary enterocytes (Morel et al., 2004; Xiao et al., 2019). *In vivo*, intestinal ApoB48 production does not appear to be altered as a result of short-term or long-term feeding of dietary lipids; instead, it has been demonstrated that a normal ApoB48-forming gut transports absorbed dietary lipids by producing a similar number but larger CMs in response to increased consumption of dietary lipids (Hayashi et al., 1990; Davidson and Shelness, 2000). Whether newly synthesized intestinal ApoB48 is degraded when lipid levels are low and whether it involves the proteasome-mediated ERAD pathway remains elusive.

## Cm Formation and Apob Synthesis and Degradation in Wt and Apobec-1 Ko Mice

Generation of the Apobec-1 knockout (KO) mouse, which is incapable of ApoB48 synthesis, has offered a unique opportunity to study the importance of ApoB48 through comparison of CM formation and lipid transport in WT animals versus the Apobec-1 KO animals with exclusive ApoB100 production (Hirano et al., 1996; Nakamuta et al., 1996). The Apobec-1 KO mouse model lacks functional Apobec-1 enzyme; thus, no RNA editing occurs and only ApoB100 is produced, including within enterocytes (Hirano et al., 1996; Nakamuta et al., 1996). Apobec-1 KO mice have comparable fat absorption, body weight gain, and food intake to WT control mice when they are maintained on a chow or high fat diet (Yao et al., 1997; Xie et al., 2003). Previous studies revealed that enterocytes isolated from Apobec-1 KO mice synthesized comparable levels of intestinal ApoB100 (as compared to ApoB48 of WT enterocytes), but the Apobec-1 KO mice had reduced secretion of CMs in a fed condition in comparison to WT mice, possibly due to the inability to efficiently assemble TG into CMs (Kendrick et al., 2001; Lo et al., 2008).

**Figure 1:**
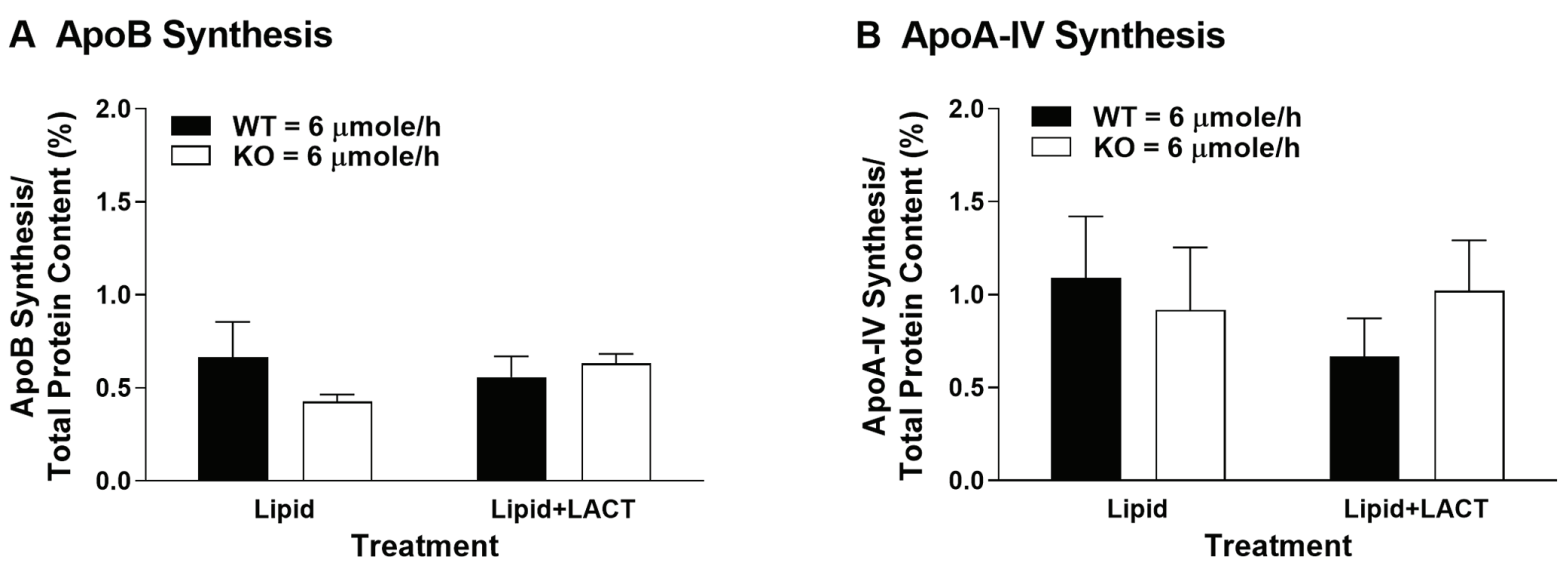
Apolipoprotein (Apo) B **(A)** and ApoA-IV **(B)** protein synthesis in wildtype (WT) and Apobec-1 knockout (KO) mice. Chow-fed WT and Apobec-1 KO mice received an intraduodenal infusion of lipid emulsion with 6 μmoles/h of triolein for 4 h, then a 7.5 cm-segment of proximal jejunum isolated by ligature in anesthetized mice was incubated with 100 μl of saline mixture containing ^3^H-leucine (200 μCi per mouse) in the absence (Lipid) or presence of lactacystin (10 μM; Lipid + LACT) based on published protocols (Kalogeris et al., 1994; Liao and Chan, 2000). After 10 min of incubation, the segment was removed and washed with saline, and mucosa of the segment was scraped, homogenized in lysis buffer, and centrifuged. For total protein content of newly synthesized proteins, precipitation of 25 μl aliquots of the cytosolic homogenate was performed with trichloroacetic acid (TCA). For synthesis of ApoB and ApoA-IV, 25 μl aliquots of the cytosolic homogenate were electrophoresed through a 4–20% gradient acrylamide SDS gel (Bio-Rad Laboratories, Hercules, CA), the gel stained with Coomassie Blue and the band of ApoB or ApoA-IV excised. Radioactive amounts of the total protein precipitates as well as the bands of ApoB and ApoA-IV were determined using scintillation counting. Synthesis of ApoB and ApoA-IV was calculated by radioactive amount of ApoB or ApoA-IV divided by radioactive amount of total protein content and multiplied by 100 (%); since ApoB100 has twice the number of leucines for labeling in comparison to ApoB48 (525 vs. 261, respectively), the radioactive values for ApoB100 were divided by two before dividing by radioactive amount of total protein content in order to be able to compare the ApoB results on a per molecule basis. Data are expressed as mean ± SEM for seven or eight animals per group. Statistical significance was assessed by two-way ANOVA (no significant differences were seen at alpha = 0.05).

To investigate whether Apobec-1 KO mice synthesized less ApoB100 *in vivo* within the small intestine, accounting for the decreased secretion of TG-rich CMs observed in our previous report (Lo et al., 2008), we measured protein synthesis in the proximal jejunum, which is a major site for synthesis of ApoB in the presence of dietary lipids (Doi et al., 2001). As seen *in vitro* (Kendrick et al., 2001), synthesis of ApoB and ApoA-IV did not differ between WT and Apobec-1 KO mice in the presence of a moderate level of dietary lipids [6 μmoles/h of triolein, previously referred to as high dose (Lo et al., 2008); left portion of Figures 1A,B]. To investigate the influence of proteasomal degradation on synthesis of ApoB and ApoA-IV, protein degradation was inhibited by lactacystin (Oda et al., 1996), and protein synthesis again assessed. Once again, synthesis of the proteins did not differ between WT and Apobec-1 KO mice (right portion of Figures 1A,B). The findings suggest that (1) the decreased secretion of TG-rich CMs observed in Apobec-1 KO mice in our previous report (Lo et al., 2008) cannot be accounted for by a decrease in ApoB100 synthesis, therefore bolstering the idea that CM formation is instead less efficient with ApoB100 (Kendrick et al., 2001); (2) degradation of intestinal ApoB48 and ApoB100 does not appear to occur *via* the proteasome pathway as seen for hepatic ApoB100 (Ciechanover, 1994; Sakata et al., 2001), but the current findings are in agreement with previous *in vitro* findings for ApoB48 (Liao and Chan, 2000; Dixon et al., 2002); and (3) ApoA-IV synthesis in Apobec1 KO mice was comparable to WT mice in the present study, and intestinal synthesis of ApoA-IV in WT and Apobec-1 KO mice was not regulated by the proteasomal process, in agreement with previous reports (Xie et al., 2003; Lo et al., 2008). Although ApoA-IV has been shown to physically interact with ApoB to modulate the process of assembly and secretion of CMs in the ER and Golgi (Lu et al., 2002; Weinberg et al., 2012), its regulation does not appear to be due to changes in synthesis or proteasomal degradation. The utility and mechanisms of intracellular degradation of ApoB48 and ApoA-IV in the small intestine remain unknown, and involvement of other degradation pathways need to be explored.

## Lipid Transport in Apobec-1 Ko and Wt Mice

**Figure 2:**
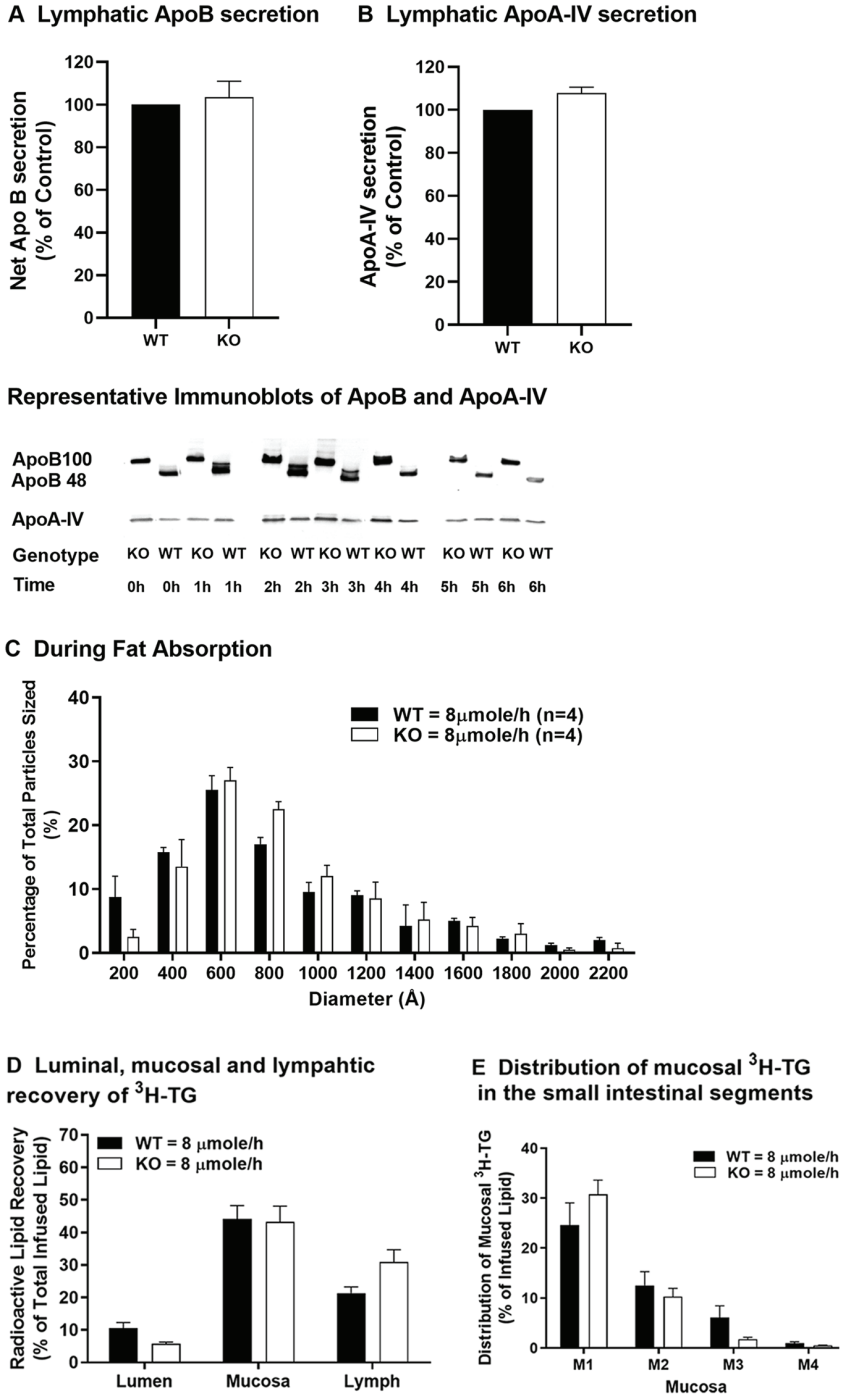
Lipid transport in WT and Apobec-1 KO mice. Chow-fed WT and Apobec-1 KO mice with lymphatic cannulation received an intraduodenal infusion of lipid emulsion containing ^3^H-triolein at a constant rate of 8 μmole/h for 6 h based on our published protocol (Lo et al., 2008). Hourly lymph was collected during lipid infusion and lymphatic levels of ApoB protein **(A)** and ApoA-IV protein **(B)** secreted over the 6-h infusion were analyzed as previously described (Lo et al., 2008). Representative immunoblots of ApoB and ApoA-IV in mice are included in the figure. Size distribution of lymphatic particles **(C)** collected from three mice of each group during the 3–4 h of intraduodenal lipid infusion at 8 μmole/h was determined by staining with 2% phosphotungstic acid and observation by transmission electron microscopy; the histogram represents the size distribution of 800 particles per mouse. At the end of the experiment, luminal contents and small intestinal mucosa were collected. Radioactive TG level determined by scintillation counting, divided by total amount of radioactive TG infused and multiplied by 100 (%) in the lumen, mucosa, and lymph (calculated from an equation that multiplied radioactive amount of hourly lymphatic TG and hourly flow rate) **(D)** and in the different segments of mucosa **(E)** are shown. Data are expressed as mean ± SEM for eight animals per group. No significant differences were observed.

In an early study utilizing blockade of plasma lipoprotein degradation, it was shown that fat-fed Apobec-1 KO mice produced larger plasma lipoproteins with greater amounts of TG in CMs than WT mice (Kendrick et al., 2001). Since plasma lipoproteins can come from the intestine or the liver, assessing lymphatic lipid transport using a lymph fistula mouse model provides a more direct and detailed study of intestinal lipid transport than does a blockade of plasma CM degradation (Lo et al., 2008). For clarity, the nomenclature of modest and high used in our previous publication to reference doses of 4 and 6 μmoles/h of triolein, respectively (Lo et al., 2008), has been changed to low and moderate (4 and 6 μmoles/h of triolein, respectively) with the addition of results for a dose of 8 μmoles/h of triolein, which we now refer to as high. Using Apobec-1 KO and WT mice and the lymph fistula model, we demonstrated that Apobec-1 KO mice, when receiving intraduodenal infusion of a lipid emulsion at a low dose (4 μmoles/h of triolein), exhibit comparable TG transport from the small intestine to the lymph in comparison to WT mice, but produce smaller CMs and have increased accumulation of TG in the mucosa of the small intestine (Lo et al., 2008). When infused with lipid emulsion at a moderate dose (6 μmoles/h of triolein), Apobec-1 KO mice secrete fewer CMs (based on reduced ApoB amount in lymph), but of comparable size to WT, and transport significantly less TG to lymph, leading to even more mucosal TG accumulation (Lo et al., 2008). In response to a high dose of TG (8 μmoles/h of triolein), Apobec-1 KO mice secrete similar amounts, relative to WT mice, of ApoB (Figure 2A) and ApoA-IV (Figure 2B) protein into the lymph. The Apobec-1 KO mice also secrete similar numbers of CM-sized particles (≥800 angstroms; ~57% of the total number of secreted particles) in comparison with WT mice (~51% of the total number of secreted particles; Figure 2C). In addition, the Apobec-1 KO mice have similar lipid transport from the small intestine into lymph (Figure 2D) and TG accumulation in intestinal mucosa (Figures 2D,E) in comparison to WT mice. Compared with Apobec-1 KO mice infused with TG at a moderate dose (Lo et al., 2008), it is possible that more ApoB100 is available for CM production in the small intestine in response to TG at a high dose, based on the results in the liver showing that increased lipid attenuates degradation of ApoB100 (Dixon et al., 1991). Whether increased synthesis and/or less degradation of intestinal ApoB100 occurs in Apobec-1 KO mice in response to the high dose of TG remains unknown; additional investigation into the synthesis of intestinal ApoB100 in response to increased availability of TG is required. Although ApoA-IV is incorporated into CMs within the ER and MTP expression is positively associated with ApoA-IV levels (Aalto-Setalälä et al., 1994; Pan et al., 2013), Apobec-1 KO mice have similar levels, relative to WT mice, of ApoA-IV when they receive intraduodenal infusion of TG at a moderate (Lo et al., 2008) or high dose (Figure 2D) and of MTP activity at a moderate TG dose (Lo et al., 2008); thus, ApoA-IV and MTP cannot account for altered numbers of CMs in the lymph (Lo et al., 2008). Interestingly, Apobec-1 KO mice have increased TG accumulation in the mucosa relative to WT mice, especially in the first mucosal part of the proximal jejunum (M1), when they receive intraduodenal infusion of TG at low or moderate doses (Lo et al., 2008); in contrast, they have comparable TG accumulation at a high dose (Figures 2D,E). This appears to be due to an increase in WT values between the moderate and high doses [mucosa TG value of 21.7% at the moderate dose (Lo et al., 2008) versus 44.2% at the high dose (Figure 2D)] rather than a decrease in Apobec-1 KO values [39.7% at the moderate dose (Lo et al., 2008) versus 43.3% at the high dose (Figure 2D)]. These findings suggest that, in response to a challenge of TG at low or moderate doses, ApoB48-forming (WT) intestine produces constant numbers but increasing size of CM particles to efficiently transport lipid from the small intestine to lymph. In contrast, ApoB100-forming (Apobec-1 KO) intestine produces fewer numbers of alterable sizes of CM particles to transport lipid from small intestine to lymph, resulting in lower transport of lipid to the lymph and more build-up of lipid within the enterocyte in comparison to ApoB48. At the highest dose of TG challenge, both ApoB48-forming (WT) and ApoB100-forming (Apobec-1 KO) intestines appear to be overwhelmed by the TG dose, secreting similar numbers and sizes of CMs and similar amounts of TG into the lymph and retaining similar amounts of TG within the mucosa. Thus, in agreement with the conclusions of an earlier study (Kendrick et al., 2001), our published results (Lo et al., 2008) and results presented here support the idea that production of the smaller ApoB48 seems to provide intestinal enterocytes with an advantage over ApoB100 in terms of the decreased energy and resources needed for ApoB48 synthesis and for its superiority to take up and transport dietary lipids into lymph, especially at low to moderate lipid levels.

## Final Thoughts and Future Directions

Dietary lipids are absorbed by intestinal enterocytes, and the transport of long-chain fatty acids from the small intestine to circulation is controlled primarily by a constant number of ApoB48-containing lipoprotein particles with alterable particle size (Hayashi et al., 1990). Insulin resistance is positively associated with increased plasma levels of ApoB48-containing lipoproteins, possibly resulting from increased intestinal CM production or impaired hepatic clearance of CM in the circulation (Dash et al., 2015). Increased stability of ApoB48, lipid availability for TG synthesis, and MTP expression for the CM assembly and production are linked with elevation of CM production in insulin resistant human subjects and animals (Lewis et al., 2004; Drouin-Chartier et al., 2018; Xiao et al., 2019). The studies of intestinal ApoB48 discussed in this perspective were primarily performed in lean mice; experiments investigating synthesis and degradation of ApoB48 and size and composition of CM particles in obese or insulin-resistant mice and humans need to be performed to determine the role of ApoB48 in controlling production and particle size of CMs and lipid transport from the gut in the face of insulin resistance.

## Data Availability Statement

The raw data supporting the conclusions of this article will be made available by the authors, without undue reservation.

## Ethics Statement

The animal study was reviewed and approved by the Institutional Animal Care and Use Committees at the University of Cincinnati. Written informed consent was obtained from the owners for the participation of their animals in this study.

## Author Contributions

CL contributed to data collection and analysis and manuscript preparation. KC contributed to data analysis and manuscript preparation. All authors contributed to the article and approved the submitted version.

## Conflict of Interest

The authors declare that the research was conducted in the absence of any commercial or financial relationships that could be construed as a potential conflict of interest.
